# Increased protein expression of ABCA1, HMG-CoA reductase, and CYP46A1 induced by garlic and allicin in the brain mouse and astrocytes-isolated from C57BL/6J

**DOI:** 10.22038/AJP.2021.17834

**Published:** 2021

**Authors:** Zahra Nazeri, Shirin Azizidoost, Maryam Cheraghzadeh, Asma Mohammadi, Alireza kheirollah

**Affiliations:** 1 *Department of Biochemistry, Medical School, Ahvaz Jundishapur University of Medical Sciences, Ahvaz, Iran*; 2 *Department of Biochemistry, Medical School, Cellular & Molecular Research Center, Medical Basic Sciences Research Institute, Ahvaz Jundishapur University of Medical Sciences, Ahvaz, Iran*

**Keywords:** Garlic, Allicin, HMG-CoA reductases, ATP binding cassette transporter 1, CYP46A1

## Abstract

**Objective:**

Regulation of cholesterol level is essential for the brain optimal function. The beneficial effect of garlic consumption on cholesterol homeostasis is well known; however, the molecular mechanism to support its properties is unclear. Here, we investigated the beneficial effect of aqueous extract of garlic and allicin on lipid profile and the main players involved in brain cholesterol homeostasis including ABCA1, HMG-CoA reductase, and CYP46A1 in both C57BL/6J mice brain and astrocytes.

**Materials and Methods:**

Thirty mice were divided into control and garlic groups. Garlic group was fed with the aqueous extract of garlic. Serum lipids were measured and brain protein levels of ABCA1, HMGCR, and CYP46A1 were determined by western blotting. Changes in these proteins expression were also studied in the presence of allicin in cultured astrocytes.

**Results:**

A moderate decrease in serum total cholesterol and a significant increase in plasma HDL-C levels (p<0.05) were detected. A significant increase in ABCA1, HMGCR, and CYP46A1 protein levels was observed in the garlic group and in the cultured astrocytes treated with allicin by western blotting (p<0.05).

**Conclusion:**

Our findings indicated that the main players involved in cholesterol turnover including HMGCR that is involved in cholesterol synthesis, ABCA1 that is important in cholesterol efflux, and CYP46A1 that is necessary in cholesterol degradation, were up regulated by garlic/allicin in both animal and cell culture model. We concluded that increasing cholesterol turnover is a possible mechanism for the beneficial effects of garlic in cholesterol homeostasis.

## Introduction

The brain is the most cholesterol-rich organ in the body. Accumulation of cholesterol as well as defects in brain cholesterol metabolism has been shown to be involved in neurodegenerative diseases like Alzheimer´s disease. So, the content of cholesterol in the brain, in particular, in elderly people, must be accurately maintained in order to keep the brain function in a steady state (Björkhem et al., 2004 ▶). Hypercholesterolemia is amongst main risk factors for atherosclerosis and subsequent heart diseases. Although the liver is considered the main source of plasma nascent high-density lipoprotein (HDL) formation and regulation of peripheral cholesterol homeostasis, maintenance of steady-state condition of brain cholesterol metabolism is also important (Zhang et al., 2015). Increased transport of cell cholesterol to apolipoproteins such as apoA1 in plasma and apoE in the brain to form HDL cholesterol and excretion of excess cholesterol by cholesterol-24 hydroxylase (CYP46A1) (Han et al., 2020 ▶) are potential mechanisms that prevent brain cholesterol deposition (Vitali et al., 2014 ▶). In the brain, CYP46A1 which is selectively expressed in neurons, converts cholesterol to 24-hydroxycholesterol that can cross the blood-brain barrier and then is cleared by the liver (Perovic et al., 2009 ▶; Lu et al., 2020 ▶).

Cellular cholesterol synthesis is a complex process which starts with mevalonate synthesis where 3-Hydroxy-3-Methylglutaryl-CoA Reductase (HMGCR) is considered the rate limiting enzyme in cholesterol synthesis. When cholesterol is synthesized in the maximum required level in astrocytes, as brain supporting cells, it should be delivered to other brain cells like neurons (Zhang et al., 2015). ATP-Binding Cassette Transporter A1 (ABCA1) is a member of the superfamily of ABC transporters that is involved in cholesterol efﬂux to apolipoprotein to form HDL (Ito et al., 2011 ▶; Chen et al., 2013 ▶). It is reported that ABCA1 is involved in macrophage cholesterol efflux to HDL for protecting macrophage foam cell from free cholesterol (Vitali et al., 2014 ▶), however, regulation of cholesterol homeostasis in the brain is poorly understood. 

Despite the known anti-hyperlipidemia medicine, owing to their related side effects and progression of drug resistance, there is an increased demand for using herbal treatments like ginger in diabetes (Azizidoost et al., 2019 ▶) or garlic in patients with hyperlipidemia or even using lipid-lowering effects of saffron-derived components in cancer therapy (Rouhi-Boroujeni et al., 2015 ▶; Hashemi et al., 2020 ▶). 

Garlic is one of the most well-known herbal medicines worldwide and there has been an increasing interest in using garlic as a cholesterol-reducing agent. Studies have shown that garlic reduces plasma lipids, particularly total cholesterol (TC), triglyceride (TG), and Low Density Lipoprotein (LDL), and also increases the high-density lipoprotein (HDL-C) level (Adler et al., 1997 ▶; Bayan et al., 2014 ▶). 

Studies have shown the effect of garlic extracts on cholesterol metabolism in the peripheral tissues (Madkor et al., 2011 ▶). *In vivo *studies have shown that garlic reduces the hepatic activity of HMGCR (Liu et al., 2002 ▶). Despite the widespread belief about hypolipidemic effect of garlic, the mechanism of this effect and its regulatory effect on the brain cholesterol homeostasis, are not understood well.

The beneficial effects of garlic consumption on cholesterol homeostasis is well known for years, however, the molecular mechanism underlying such properties is unknown. The protective effects of garlic on cardiovascular disease, cancer, and diabetes (Sarkaki et al., 2012 ▶; Hajhashemi et al., 2014 ▶) have been investigated in animal models or epidemiologic studies, but its effect on the brain cholesterol homeostasis has not yet been clarified. Since the brain is segregated from systemic circulation by the blood-brain barrier and relies on its own cholesterol, the aim of this study was to evaluate the effect of garlic extract on the protein levels of main players involved in the brain cholesterol homeostasis such as HMGCR that is involved in cholesterol synthesis, ABCA1 that is important in cholesterol efflux, and CYP46A1 that is necessary, in cholesterol degradation in the brain of C57BL/6J mice. Also, we evaluated whether allicin as the main organosulfur component of garlic has the same beneficial effect on the mentioned proteins in astrocytes which are the supporting and most abundant cells in the brain. 

## Materials and Methods

**Materials**

Fresh bulbs of garlic were purchased from markets which was collected from the local fields of Ramhormoz/Khozestan/Iran and authenticated by Department of Pharmacognosy, School of Pharmacy, Ahvaz Jondishapour University of Medical Sciences as the purple stripe hardneck garlic (Persian star). Based on the Baghalian et al report, the allicin contain of this strain is more than 4.5 mg/g of garlic (Baghalian et al., 2005). The voucher samples were preserved as reference in the Biochemistry Department of Medical School, Ahvaz Jondishapour University of Medical Sciences. Mouse anti-ABCA1 and anti-β-actin antibodies were obtained from Sigma Aldrich. Polyvinylidene fluoride (PVDF) was from Roche Applied Science, Germany. Mouse anti-CYP46A1was purchased from Santa Cruz Biotechnology, USA. Mouse Anti-HMGCR (a kind gift from Professor TY Chang, Geisel School of Medicine at Dartmouth, Hanover, USA). Allicin (CAS 539-86-6) was purchased from Santa Cruz Biotechnology, USA. All other chemicals were purchased from Merck Chemicals, Germany.

**Preparation of garlic extract**

An aqueous extract of fresh garlic was prepared using freshly peeled cloves; 50 g of the edible portion was chopped and homogenized in 100 ml of double distilled and autoclaved water in a blender. The homogenate was then filtered by passage through a nitrocellulose membrane filter diameter 25 mm, pore size 0.45 μm to give a crude aqueous extract of 500 mg of garlic/ml. The extract was collected in a sterile vial and stored at -20°C until used.

**Animals and diets**

Eight-week-old male C57BL/6J mice, weighing 25-30 g, were housed in a temperature-controlled room (24±1°C) under 12 hr light/dark conditions with free access to food and water. Animal procedures were done in accordance with the guidelines for animal care prepared by Committee on Care and Use of Laboratory Animal resources, National Research Council, USA, and were approved by the Animal Ethics Committee (IAEC) of AJUMS for the Purpose of Control and Supervision of Experiments on Animals. Every effort was made to minimize the animal suffering and decrease the number of animals used. Mice were fed with a standard and commercial chow diet and water for a week to stabilize their metabolic condition. After acclimatization, the mice were randomly allocated into 2 groups of 15 mice each, 3 to 4 mice/cages (size=L 465 mm, W 305 mm, H 160 mm), and they were administered with dH_2_O or 150 mg/kg body weight of aqueous extract of garlic, three times a week by oral gavage for six weeks. Body weight was measured once a week during the treatment period. At the end of the experiment, the mice from each group were deprived of food overnight and anesthetized with ketamine/xylazine (10/1). Blood samples were collected by cardiac puncture and centrifuged at 3000×g for 15 min at 4°C to obtain plasma, which was then stored at -20°C for biochemical analyses. The brain was dissected, frozen immediately in liquid nitrogen, and stored in -70°C. 

**Isolation, culture and treatment of astrocytes**

Astrocytes were isolated from the newborn brain of C57BL/6 mice. Briefly, after brain dissection and removal of the meninges, the brain was cut into small pieces and incubated with 0.25% trypsin and Dulbecco’s phosphate-buffered saline (DPBS) (1:1) for 3 min at 37°C. Then, the suspension was centrifuged at 1000 rpm for 1 min. The cell pellet was cultured in low-glucose DMEM containing 10% fetal bovine serum and 1% penicillin/streptomycin for a 1-week primary and a subsequent 1-week secondary culture (Kheirollah et al., 2014 ▶). Astrocytes were treated with 5 µg/ml allicin. 

**Western blotting**

The mice brain was washed with PBS, and homogenized by sonication in ice-cold RIPA buffer with protease inhibitor cocktail. Also, astrocytes were harvested with ice-cold RIPA buffer with protease inhibitor cocktail. Total protein concentrations were determined using the Lowry method (Lowry et al., 1951 ▶), and 100 μg of protein from each sample was loaded onto sodium dodecyl sulfate polyacrylamide gel electrophoresis (SDS-PAGE) (8% polyacrylamide gel). Proteins were transferred onto PVDF membrane. Blocking was performed with 5% skimmed milk in TBS containing 0.1% tween 20 to prevent nonspecific binding. Immunoblotting was performed using anti-ABCA1, anti-CYP46A1, anti-HMGCR, or β-actin antibodies. The immunoblots were visualized using a dilution of horseradish peroxidase-conjugated anti-rabbit secondary antibody and chemiluminescence western blotting substrate. Densitometric analysis was carried out using ImageJ software. 

**Lipid profile**

Aqueous extract of garlic was given to the garlic group and an equivalent amount of dH_2_O to the control group. After 6 weeks, mice were anesthetized and blood was collected from their heart. The level of TC, TG, HDL-C, and LDL-C was evaluated by enzymatic colorimetric assays. 

**Statistical analysis**

SPSS software V 15.0 was used for all statistical analyses. The effect of aqueous garlic extract on plasma lipid profile was analyzed and the data is presented as mean±SEM. Statistical significance of the differences between the two groups was analyzed using t-test. The level of significance was set as p<0.05. All experiments were done in triplicate.

## Results

**Garlic has beneficial effects on lipid profile**

To confirm the previous reports (Zeb et al., 2018; Shabani et al., 2019; Sangouni et al., 2020), serum lipid profile in our animal model was checked and presented in table 1. The significant changes were found in the serum lipid profile in the garlic group compared to the control group. In the garlic group, the HDL-C showed a 35.04% increased (p<0.05) while the LDL-C and TG levels decreased significantly by 50.55 (p<0.01) and 26.82% (p<0.05), respectively (Table 1).

**Table 1:** Effects of aqueous extract of garlic on plasma lipid profile

Plasma	Control	Garlic
Total cholesterol (mg/dl)	153.18±5.78	135.50±7.38
Triglyceride (mg/dl)	181.18±11.9	132.58±7.95*
HDL cholesterol (mg/dl)	58.39±8.32	78.85±4.91*
LDL cholesterol (mg/dl)	57.90±8.11	28.63±5.55**

Values are expressed as mean±SEM; n=15 in each group. *Significantly different from the control group at *p<0.05, **p<0.01 as examined by Student’s t-test.

**Effect of garlic on some main players of cholesterol homeostasis in the mouse brain**

**Figure 1:**
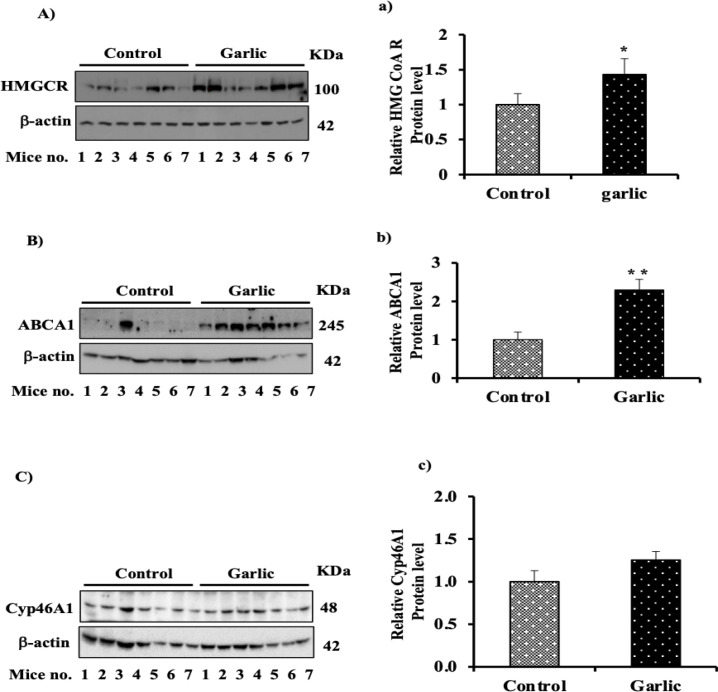
Effect of garlic on protein expression of HMGCR, ABCA1 and CYP46A1 in mouse brain tissue. Mice were randomly allocated into 2 groups of 15 mice each and were administered with dH_2_O or 150 mg/kg/weight aqueous extract of garlic, three times a week by oral gavage, for six weeks. At the end of the experiment, the mice were anesthetized and the brain was dissected, frozen immediately in liquid nitrogen, and stored in −70°C. Protein expression pattern of (A) HMG-CoA reductase, (B) ABCA1 (because of abnormal and the huge difference between ABCA1 band related to the third sample in the control group, it was excluded when plotting the quantitative data of imageJ presented in Figure 1B), and (C) CYP46A1 in the brain of control and garlic group was analyzed by western blotting. Equal loading was assessed with an anti β-actin antibody. Bar graph represents quantification of Western blots after normalizing against β-actin for a) HMGCAR, b) ABCA1, and c) CYP46A1. The mean±SEM of three independent experiments is shown. ^*^p<0.05 and^ **^p<0.01 present significant differences from the control group

The regulatory effect of garlic on HMGCR protein level was analyzed by SDS-PAGE and western blotting. Treatment with aqueous garlic extract for six weeks caused a significant increase of HMGCR in the mice brain by 30% (p< 0.05) (Figure 1A), indicating the effect of garlic on the rate-limiting enzyme in the biosynthesis of cholesterol. As expected, variations in protein expression in different lanes for both control and garlic-treated group were observed for all proteins, showing each mouse has its own pattern or response, which could be different from others even in the same group. 

Effect of garlic on mice brain ABCA1, as one of the main elements involved in cholesterol efflux, was analyzed after feeding mice with garlic. Data presented in Figure 1B indicate that garlic caused an approximately 90% (p<0.01) increase in ABCA1 protein content in the garlic-treated group compared to the control group. 

To check the effect of garlic on brain CYP46A1 protein content, brain homogenate was subjected to SDS-PAGE and western blotting. Although garlic leads to an enhancement of CYP46A1 protein level by 28%, no significant difference was observed compared to the control group (Figure 1C).

**Allicin shows effects similar to garlic on protein expression of HMGCR, ABCA1 and CYP46A1**

**Figure 2:**
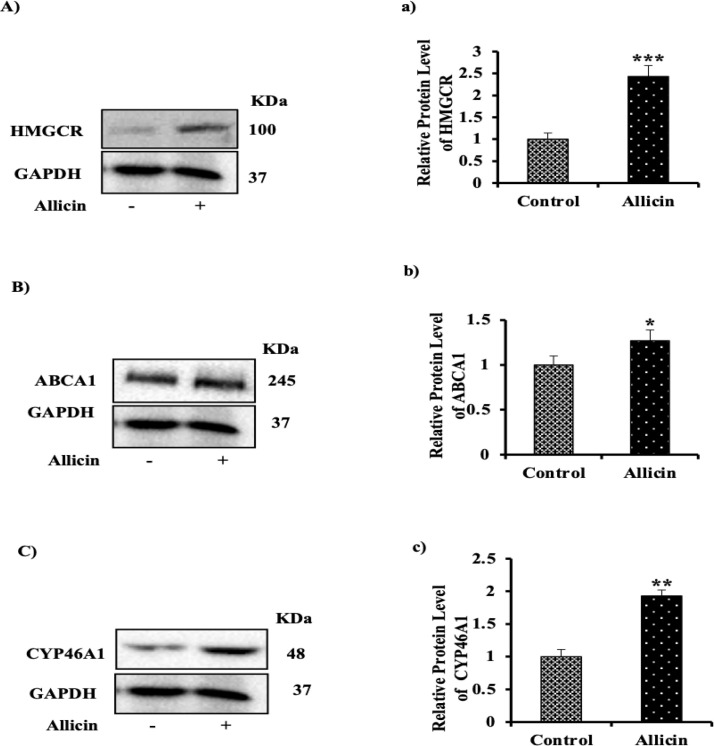
Effect of allicin on protein expression of HMGCR, ABCA1 and CYP46A1 in astrocytes isolated from C57BL/6J. Astrocytes were isolated and cultured in low-glucose DMEM containing 10% fetal bovine serum and 1% penicillin/streptomycin for a 1-week primary and a subsequent one-week secondary culture. Astrocytes were treated with 5 µg/ml allicin and after incubation time, cells were harvested and analyzed by western blotting by using specific antibody against (A) HMG-CoA reductase, (B) ABCA1, and (C) CYP46A1. Equal loading was assessed with an anti β-actin antibody. Bar graph represents quantification of Western blots after normalizing against GAPDH for a) HMGCAR, b) ABCA1, and c) CYP46A1. The mean±SEM of three independent experiments is shown. ^*^p<0.05, ^**^p<0.01, and ^***^p<0.005 present significant differences from the control group

To check the effect of allicin on HMGCR, ABCA1 and CYP46A1 content in astrocytes, the cell lysate was subjected to SDS-PAGE and western blotting. Allicin treatment caused a significant increase of HMGCR (Figure 2A), ABCA1 (Figure 2B) and CYP46A1 (Figure 2C) protein levels compared to the control group. It can be concluded that the effect of garlic on the main players in brain cholesterol homeostasis could be most likely attributed to allicin.

## Discussion

The present study shows anti-cholesterolemic effects of aqueous extract of garlic and its main organosulfur component, allicin. The major findings are as follows: (1) Garlic has beneficial effects on lipid profile by inducing HDL generation and lowering total cholesterol, LDL-C, and TG in blood circulation; (2) Garlic increases brain HMG-CoA reductase, ABCA1, and CYP46A1 protein levels in the mouse brain; and (3) allicin has the same effects as garlic on increasing HMGCR, ABCA1 and CYP46A1 protein levels in astrocytes, as the supporting and most abundant cells in the brain. We concluded that increasing cholesterol turnover is the possible mechanism for the beneficial effects of garlic on cholesterol homeostasis and allicin most likely contributed to the garlic beneficial effects. 

The brain cells are segregated from systemic circulation by the blood-brain barrier, therefore they rely on their own cholesterol biosynthesis. Astrocytes, as the main source of brain HDL for supporting nerves, was used to assess the effect of garlic/allicin on HMGCR/ABCA1/CYP46A1 pathway, which includes the most important players involved in cholesterol turnover in the brain. We found that HMGCR, as the crucial rate limiting enzyme in cholesterol synthesis, ABCA1 that is important in cholesterol efflux, and CYP46A1 that is necessary in cholesterol degradation are upregulated by both aqueous extract of garlic and allicin. The effect of garlic/allicin should therefore be on entire cholesterol turnover pathway including its synthesis, trafficking, and degradation. 

HMG-CoA reductase is the first committed step of the mevalonate pathway, involved in the synthesis of sterols and isoprenoids that are very important and crucial components of the brain health and its optimal function. In addition to all necessary mevalonate pathway intermediates, cholesterol serves as the precursor for synthesis of oxysterols that are suggested to play various roles in central nervous system (CNS). With this background and as a result, cholesterol turnover could be managed by garlic or allicin in the brain or astrocytes by upregulation of HMGCR and CYP46A1. Of course other enzymes involved in the brain cholesterol homeostasis should be considered before come to further conclusions.

Since the amount of HMG-CoA reductase is controlled by PPAR gamma, upregulation of this enzyme in our experiment following treatment with both allicin and aqueous extract of garlic might be dependent on PPAR gamma activation. Results of Wang et al indicated that alliin, the allicin precursor, increased the expression and activation of PPAR gamma in BALB/c mice lung (Wang et al., 2017 ▶). Therefore, increasing HMG-CoA reductase observed in our study could be induced through activating PPAR gamma. 

Our HMGCR results are in contrast to the findings of Liu et al. who reported unchanged protein level of HMGCR in cultured rat hepatocytes following treatment with water-soluble organosulfur compounds of garlic (Liu and Yeh, 2001 ▶; Liu and Yeh, 2002 ▶). This may be due to the fact that regulation of this protein in the brain is different from its regulation in the peripheral tissue.

Studies have shown that there is a reverse correlation between plasma HDL level and risk of cardiovascular diseases, for instance the recent study of Morze et al. 2020 ▶. So, it can be implied that factors related to the HDL formation can be atheroprotective by eliminating excess cholesterol from arterial cells (Rothblat et al., 1999 ▶; Kudinov et al., 2020 ▶). In the brain, HDL generation is very important for delivery of cholesterol from astrocytes to neurons and ABCA1 plays a critical role in HDL production (Kennedy et al., 2005 ▶; Kheirollah et al., 2014 ▶). Previous studies show that ABCA1 is a key element in reverse cholesterol transport to form HDL-cholesterol (Kheirollah et al., 2006 ▶; Ito et al., 2011 ▶). Increased protein levels of ABCA1 in THP-1 macrophages treated with allicin have validated our findings about regulatory effects of garlic and allicin on ABCA1 which has a crucial role in cholesterol homeostasis (Malekpour‐Dehkordi et al., 2013 ▶; Lin et al., 2017 ▶). 

In the present study, significant increases of both HMGCR and ABCA1 protein level by both aqueous extract of garlic and allicin may participate in brain HDL formation. It can be concluded that increased level of ABCA1 may be a regulatory compensatory mechanism of brain cholesterol metabolism in response to increased level of HMGCR to efflux cholesterol to apolipoprotein for HDL generation. It can be suggested that as like as the atheroprotective effect, garlic has the same effect in the central nervous system (CNS) by increasing the level of main proteins involved in HDL formation. 

In addition, it has been reported that livers X receptors (LXRs), including LXRα and LXRβ, belonging to the nuclear receptor superfamily control the reverse cholesterol transport via regulation of ABC genes such as ABCA1 (Repa et al., 2002 ▶; Yang et al., 2020 ▶). Garlic induced LXRα expression and activation in the intestine (Mohammadi and Oshaghi, 2014 ▶). Garlic component(s) that activate or modulate LXRs may induce ABCA1 expression (Cui et al., 2017 ▶) in the brain through up-regulation of LXRs. It is likely that some garlic components serve as a LXRs agonist, however, more studies are needed to test this hypothesis.

Here, we also reported an increased CYP46A1 protein level in astrocytes treated with allicin. CYP46A1 (Saadane et al., 2019 ▶; Han et al., 2020 ▶) is involved in converting cholesterol into a water-soluble metabolite, 24S-OH cholesterol, to increase its solubility for excretion from the CNS. Previous study has demonstrated that CYP46A1 expression is under control of Specificity proteins (Sp protein) family and interestingly LXR targets those genes contributing to Sp binding elements in the CYP46A1 promoter (Djelti et al., 2015 ▶). Potentially, garlic serves as an LXR element that has a cross-talk with Sp binding sites in CYP46A1 promoter and so leads to increased CYP46A1 expression in the brain.

As a general result, we think that garlic consumption increases cholesterol synthesis, cholesterol efflux, and cholesterol degradation respectively through affecting HMGCR, ABCA1, and CYP46A1, thereby increasing the cholesterol turnover through the mevalonate pathway. Increasing the mevalonate pathway has many beneficial effects on the brain by activating more than 35 enzymes (Kotti et al., 2006 ▶; Moutinho et al., 2017 ▶) in this pathway and generating many intermediate substances which are essential for the brain health and functions, such as nonsterol isoprenoids or oxysterols like lanosterol, which play an important role in learning.

In conclusion, garlic as a food supplement has a lipid-lowering effect. There is no solid evidence for the effect of garlic on the players involved in the brain cholesterol homeostasis or for allicin on cultured astrocytes so far, but our data show that garlic has beneficial effects on cholesterol not only in peripheral tissue but also in the brain as an organ with a high cholesterol content in the body.

According to this study, because garlic especially its organosulfur component, allicin, has a regulatory effect on the proteins involved in cholesterol turnover, we suggest a daily garlic consumption in particular in elderly diet.

## References

[B1] Adler AJ, Holub BJ (1997). Effect of garlic and fish-oil supplementation on serum lipid and lipoprotein concentrations in hypercholesterolemic men. Am J Clin Nutr.

[B2] Azizidoost S, Nazeri Z, Mohammadi A, Mohammadzadeh G, Cheraghzadeh M, Jafari A, Kheirollah A (2019). Effect of hydroalcoholic ginger extract on brain HMG-CoA reductase and CYP46A1 levels in streptozotocin-induced diabetic rats. Avicenna J Med Biotechnol.

[B3] Bayan L, Koulivand PH, Gorji A (2014). Garlic: a review of potential therapeutic effects. Avicenna J Phytomed.

[B4] Björkhem I, Meaney S (2004). Brain cholesterol: long secret life behind a barrier. ATVB.

[B5] Chen J, Zhang X, Kusumo H, Costa LG, Guizzetti M (2013). Cholesterol efflux is differentially regulated in neurons and astrocytes: implications for brain cholesterol homeostasis. Biochim Biophys Acta.

[B6] Cui X, Chopp M, Zhang Z, Li R, Zacharek A, Landschoot-Ward J, Venkat P, Chen J (2017). ABCA1/ApoE/HDL pathway mediates GW3965-induced neurorestoration after stroke. Stroke.

[B7] De Martino A, Filomeni G, Aquilano K, Ciriolo MR, Rotilio G (2006). Effects of water garlic extracts on cell cycle and viability of HepG2 hepatoma cells. J Nutr Biochem.

[B8] Djelti F, Braudeau J, Hudry E, Dhenain M, Varin J, Bièche I, Marquer C, Chali F, Ayciriex S, Auzeil N, Alves S, Langui D, Potier MC, Laprevote O, Vidaud M, Duyckaerts C, Miles R, Aubourg P, Cartier N (2015). CYP46A1 inhibition, brain cholesterol accumulation and neurodegeneration pave the way for Alzheimer’s disease. Brain.

[B9] Hajhashemi V, Dashti G, Saberi S, Malekjamshidi P (2014). The effect of hydroalcoholic extract and essential oil of Heracleum persicum on lipid profile in cholesterol-fed rabbits. Avicenna J Phytomed.

[B10] Han M, Wang S, Yang N, Wang X, Zhao W, Saed HS, Daubon T, Huang B, Chen A, Li G, Miletic H, Thorsen F, Bjerkvig R, Li X, Wang J (2020). Therapeutic implications of altered cholesterol homeostasis mediated by loss of CYP46A1 in human glioblastoma. EMBO Mol Med.

[B11] Hashemi SA, Bathaie Sz, Mohagheghi MA (2020). Crocetin and crocin decreased cholesterol and triglyceride content of both breast cancer tumors and cell lines. Avicenna J Phytomed.

[B12] Ito J, Nagayasu Y, Kheirollah A, Abe-Dohmae S, Yokoyama S (2011). ApoA-I enhances generation of HDL-like lipoproteins through interaction between ABCA1 and phospholipase Cgamma in rat astrocytes. Biochim Biophys Acta.

[B13] Kennedy MA, Barrera GC, Nakamura K, Baldán A, Tarr P, Fishbein MC, Frank J, Francone OL, Edwards PA (2005). ABCG1 has a critical role in mediating cholesterol efflux to HDL and preventing cellular lipid accumulation. Cell Metab.

[B14] Kheirollah A, Ito J-i, Nagayasu Y, Lu R, Yokoyama S (2006). Cyclosporin A inhibits apolipoprotein AI-induced early events in cellular cholesterol homeostasis in rat astrocytes. Neuropharmacology.

[B15] Kheirollah A, Nagayasu Y, Ueda H, Yokoyama S, Michikawa M, Ito J (2014). Involvement of cdc42/Rho kinase in apoA-I-mediated cholesterol efflux through interaction between cytosolic lipid-protein particles and microtubules in rat astrocytes. J Neurosci Res.

[B16] Kotti TJ, Ramirez DM, Pfeiffer BE, Huber KM, Russell DW (2006). Brain cholesterol turnover required for geranylgeraniol production and learning in mice. Proc Natl Acad Sci U S A.

[B17] Kudinov VA, Alekseeva OY, Torkhovskaya TI, Baskaev KK, Artyushev RI, Saburina IN, Markin SS (2020). High-density lipoproteins as homeostatic nanoparticles of blood plasma. Int J Mol Sci.

[B18] Lin XL, Hu HJ, Liu YB, Hu XM, Fan XJ, Zou WW, Pan YQ, Zhou WQ, Peng MW, Gu CH (2017). Allicin induces the upregulation of ABCA1 expression via PPARgamma/LXRalpha signaling in THP-1 macrophage-derived foam cells. Int J Mol Med.

[B19] Liu L, Yeh Y-Y (2002). S-alk (en) yl cysteines of garlic inhibit cholesterol synthesis by deactivating HMG-CoA reductase in cultured rat hepatocytes. J nutr.

[B20] Liu L, Yeh YY (2001). Water‐soluble organosulfur compounds of garlic inhibit fatty acid and triglyceride syntheses in cultured rat hepatocytes. Lipids.

[B21] Lowry OH, Rosebrough NJ, Farr AL, Randall RJ (1951). Protein measurement with the Folin phenol reagent. JBC.

[B22] Lu F, Fan S, Romo AR, Xu D, Ferriero DM, Jiang X (2020). Serum 24S-hydroxycholesterol predicts long-term brain structural and functional outcomes after hypoxia-ischemia in neonatal mice. J Cereb Blood Flow Metab.

[B23] Madkor HR, Mansour SW, Ramadan G (2011). Modulatory effects of garlic, ginger, turmeric and their mixture on hyperglycaemia, dyslipidaemia and oxidative stress in streptozotocin–nicotinamide diabetic rats. Br J Nutr.

[B24] Malekpour-Dehkordi Z, Javadi E, Doosti M, Paknejad M, Nourbakhsh M, Yassa N, Gerayesh-Nejad S, Heshmat R (2013). S‐Allylcysteine, a garlic compound, increases ABCA1 expression in human THP‐1 macrophages. Phytother Res.

[B25] Mohammadi A, Oshaghi EA (2014). Effect of garlic on lipid profile and expression of LXR alpha in intestine and liver of hypercholesterolemic mice. J Diabetes Metab Disord.

[B26] Morze J, Koch M, Aroner SA, Budoff M, McClelland RL, Mukamal KJ, Jensen MK (2020). Associations of HDL subspecies defined by ApoC3 with non-alcoholic fatty liver disease: The multi-ethnic study of atherosclerosis. J Clin Med.

[B27] Moutinho M, Nunes MJ, Rodrigues E (2017). The mevalonate pathway in neurons: It's not just about cholesterol. Exp Cell Res.

[B28] Perovic M, Mladenovic Djordjevic A, Smiljanic K, Tanic N, Rakic L, Ruzdijic S, Kanazir S (2009). Expression of cholesterol homeostasis genes in the brain of the male rat is affected by age and dietary restriction. Biogerontology.

[B29] Repa JJ, Berge KE, Pomajzl C, Richardson JA, Hobbs HH, Mangelsdorf DJ (2002). Regulation of ATP-binding cassette sterol transporters, ABCG5 and ABCG8, by the oxysterol receptors, LXRα and β. JBC.

[B30] Rothblat GH, de la Llera-Moya M, Atger V, Kellner-Weibel G, Williams DL, Phillips MC (1999). Cell cholesterol efflux: integration of old and new observations provides new insights. JLR.

[B31] Rouhi-Boroujeni H, Rouhi-Boroujeni H, Heidarian E, Mohammadizadeh F, Rafieian-Kopaei M (2015). Herbs with anti-lipid effects and their interactions with statins as a chemical anti-hyperlipidemia group drugs: A systematic review. ARYA atherosclerosis.

[B32] Saadane A, Mast N, Trichonas G, Chakraborty D, Hammer S, Busik JV, Grant MB, Pikuleva IA (2019). Retinal vascular abnormalities and microglia activation in mice with deficiency in cytochrome P450 46A1-mediated cholesterol removal. Am J Pathol.

[B33] Sangouni AA, Mohammad Hosseini Azar MR, Alizadeh M (2020). Effect of garlic powder supplementation on hepatic steatosis, liver enzymes and lipid profile in patients with non-alcoholic fatty liver disease: a double-blind randomised controlled clinical trial. Br J Nutr.

[B34] Sarkaki A, Valipour Chehardacheric S, Farbood Y, Mansouri SMT, Naghizadeh B, Basirian E (2012). Effects of fresh, aged and cooked garlic extracts on short- and long-term memory in diabetic rats. Avicenna J Phytomed.

[B35] Shabani E, Sayemiri K, Mohammadpour M (2019). The effect of garlic on lipid profile and glucose parameters in diabetic patients: A systematic review and meta-analysis. Prim Care Diabetes.

[B36] Vitali C, Wellington CL, Calabresi L (2014). HDL and cholesterol handling in the brain. Cardiovasc Res.

[B37] Wang YL, Guo XY, He W, Chen RJ, Zhuang R (2017). Effects of alliin on LPS-induced acute lung injury by activating PPARgamma. Microb Pathog.

[B38] Yang R, Zhao Y, Gu Y, Yang Y, Gao X, Yuan Y, Xiao L, Zhang J, Sun C, Yang H, Qin J, Li J, Zhang F, Zhang L, Ye J (2020). Isocitrate dehydrogenase 1 mutation enhances 24(S)-hydroxycholesterol production and alters cholesterol homeostasis in glioma. Oncogene.

[B39] Zeb F, Safdar M, Fatima S, Khan S, Alam S, Muhammad M, Syed A, Habib F, Shakoor H (2018). Supplementation of garlic and coriander seed powder: Impact on body mass index, lipid profile and blood pressure of hyperlipidemic patients. Pak J Pharm Sci.

[B40] Zhang J, Liu Q (2015). Cholesterol metabolism and homeostasis in the brain. Protein & cell.

